# Assessing the Carasau Bread Doughs Microwave Spectra

**DOI:** 10.3390/foods14071177

**Published:** 2025-03-27

**Authors:** Elisabetta Orrù, Matteo B. Lodi, Luca Lodi

**Affiliations:** 1 Department of Electrical and Electronic Engineering, University of Cagliari, Via Marengo 2, 09123 Cagliari, Italy; 2 Department of Economics and Management, Università degli Studi di Firenze, Via delle Pandette 32, 50127 Firenze, Italy; luca.lodi@unifi.it

**Keywords:** bread doughs, Carasau, food engineering, microwave spectroscopy

## Abstract

Carasau bread (CB) is a traditional Sardinian flatbread with significant market potential, driving the need for advanced quality monitoring solutions in its production. Recent advancements in automation and engineering have enhanced process control, but a comprehensive understanding of CB dough properties remains essential. Dielectric spectroscopy (DS), particularly in the microwave (MW) range, has emerged as a non-destructive, cost-effective tool for food characterization, providing insights into microstructure and composition. MW DS has been applied to assess fermentation dynamics and ingredient influence in CB doughs, with previous studies modeling dielectric properties using a third-order Cole–Cole model up to 8.5 GHz and later extending to 20 GHz. Despite these advancements, the repeatability, reliability, and consistency of MW DS measurements on CB doughs have not been systematically assessed. This study aims to fill this gap by analyzing MW DS measurements on ten CB dough samples with standard composition (water 50%, yeast 1.5%, salt 1.5%) in the 0.5–6 GHz range, both before and after leavening, for 10 different samples and a total of 100 measurements. Even though the correlation between spectra is high, and even if the coefficient of variation is below 5% before leavening, the z-score analysis and the kernel density estimation highlighted that the distribution of dielectric data is heterogeneous, showing that variability across samples exists, especially after leavening. Finally, the influence of pressure, temperature, and relative humidity was excluded. This statistical evaluation of MW DS measurement provided critical insights into the robustness of MW DS for industrial applications.

## 1. Introduction

Carasau bread (CB), or “pane carasau”, is a traditional flatbread from Sardinia, Italy [1]. CB shares similarities with other Mediterranean flatbreads [1]. CB is characterized by a crisp texture and a circular shape (18–40 cm in diameter). CB is made from re-milled durum wheat semolina, de-ionized water, iodized salt, and baker’s yeast [2]. The production process involves mixing, fermentation, sheeting, leavening, and a two-step baking procedure at high temperatures (~570 °C and ~400 °C) [2]. Once cooled, CB is packaged and distributed [2].

With its sustainable nature—requiring minimal water and no tableware—and its association with Sardinia’s longevity-linked dietary habits [3], CB has gained increasing popularity, even beyond Italy, with significant market growth potential. The industry surrounding its production is undergoing a transformation driven by digitalization and automation, necessitating advanced quality monitoring solutions throughout the manufacturing process [2]. The industry has responded to this demand with advancements in automation and engineering, including the introduction of hybrid Petri net models and wireless sensor networks to monitor processing and environmental parameters [4]. However, recent studies emphasize that engineering tools alone are insufficient; a comprehensive, multifactorial approach is needed to integrate and interpret process and environmental data to optimize production quality and efficiency.

Expanding knowledge of the physical properties of CB doughs is essential for developing new tools and devices to enhance production. Traditionally, texture analysis is performed, but, even though it is fast and not costly, it is limited in providing complete information on a bread dough’s physical properties [5,6,7,8,9,10]. Therefore, various characterization methods have been employed to assess its fundamental properties and quality. Rheological studies have explored the effects of different wheat varieties, processing conditions, and composition on dough behavior [5,6]. In [5], four different semolina durum wheat and the textural (i.e., hardness and stickiness) and rheological features (i.e., consistograph and alveograph indexes) were correlated, finding that the gluten content only affects the final bread moisture content. From the perspective of traditional characterization methods, these samples are known to present stickiness ranging between 0.02 and 0.30 Ns and hardness between 0.02 and 0.1 N [5]. On the other hand, in [6], a rheometer equipped with a plate–plate fixture was used to perform frequency sweep and creep tests, finding that the addition of water significantly decreases the viscosity while improving the deformability of the dough. The samples present complex viscosity values ranging from 1000 to 10,000 Pa⋅s [6]. Additionally, thermogravimetric and calorimetric analyses have examined the impact of water, yeast, and salt content, finding that the amount of yeast and salt influences the dough network force [7]. Recent advancements include the use of low-field nuclear magnetic resonance (NMR) to study microstructural changes due to water and flour content [8]. Findings from [8] indicate that bound and free water within dough strongly affect its rheological behavior and processability, as well as its gluten network. Fourier transform infrared (FTIR) spectroscopy, focused on the Amide III band, has been correlated with rheological properties, interpreted using the weak gel framework, to model dough characteristics [9]. The β-sheets and α-helices protein conformations were found to strongly affect the gluten network’s mechanical strength [9]. On the other hand, cryogenic dielectric spectroscopy (DS), spanning 0.1 Hz to 10 MHz, has provided further insights by linking dielectric properties to rheological behavior and water content, protein amount, gluten amount, and gluten index [10]. In particular, the dielectric losses were found to be weak and negatively correlated with the total water content but positively correlated with semolina protein and gluten amount, thus suggesting that the dielectric response is mostly due to bound water [10]. Furthermore, the correlations between the rheological Burgers parameters and the dielectric ones showed that starch hydration at the glucose ring level facilitates dough compliance by easing molecular movements, while the hydroxyl groups in starch retard deformation and potentially increase viscosity, though their correlation with dielectric parameters is not statistically significant [10]. These studies, despite poorly focusing on repeatability, consistency, and reliability, contributed to a more comprehensive understanding of CB dough, supporting improvements in production and quality control. However, given limitations associated with costs, destructivity, and the need for contact with the sample, there is still a lack of cost-effective tools for characterizing the CB doughs during production.

Considering the aforementioned physical methods for the characterization of CB doughs, DS approaches constitute an interesting solution and valuable tool for industrial processing. Indeed, DS is a powerful, non-destructive technique for characterizing food materials [11]. By measuring, modeling, and analyzing the dielectric response across different frequencies, DS provides valuable insights into the microstructure and organoleptic properties of food products. MW DS has been widely applied in food analysis, including assessing apple maturity [11] and pork meat quality [12]. It has also been used to monitor the development and dynamics of the apple-candying process [13], providing insights into product quality. Beyond preliminary food characterization, MW DS enables the development of innovative applications. For example, microwave imaging systems, such as antenna arrays, are gaining attention for detecting physical contamination in the food industry [14,15,16]. Additionally, planar sensors have been shown to assess the composition of vegetable oils [17], while impedance sensors have demonstrated the ability to detect food pathogens by analyzing variations in dielectric properties [18].

Despite MW sensors and devices having been proposed as a cost-effective approach for quality assessment, a thorough understanding of the MW response of Carasau dough is essential for their effective implementation. Previous studies have investigated the dielectric properties of Carasau dough, primarily focusing on fermentation dynamics through dielectric spectroscopy [19]. An analysis up to 8.5 GHz examined samples made from different semolina batches, identifying the best MW DS model and applying a third-order Cole–Cole model to study dielectric permittivity variations during leavening, showing relevant changes associated with water content variation and gluten network formation [19]. However, while MW DS data have been quantitatively modeled, the influence of dough composition on dielectric properties remains largely unexplored. In [20], complex dielectric permittivity measurements were conducted up to 8.5 GHz, examining the influence of water content, salt, and yeast concentrations on the dielectric spectra. A third-order Cole-Cole model was employed to interpret the microwave response, achieving a maximum error of 1.58% and 1.60% for the real and imaginary parts of permittivity, respectively. Additionally, thermogravimetric analysis supported the microwave spectroscopy investigation, highlighting the strong dependence of dielectric properties on water content. The findings indicated that an increase in water content leads to a higher fraction of bound water at the expense of free water [20]. Recently, the validity of the third-order Cole-Cole model was extended up to 20 GHz [21], assessing the influence of salt, yeast, and water content in a way consistent with previous results. These methodologies and experimental results lead to the design and preliminary test of a patch antenna working at 5.8 GHz to measure wirelessly and in-line the water content of CB dough sheets [22].

Despite these MW DS findings about the fundamental properties of CB doughs and the effects of ingredients, a major knowledge gap remains. Indeed, having proved that MW DS can be used to assess the quality of CB doughs and assist in its production, the repeatability and reliability of the MW DS measurements have not been studied in a quantitative and systematic way. Building upon these insights, this study aims to analyze MW DS spectra of ten samples of CB doughs with an average composition (water 50%, yeast 1.5%, salt 1.5%) in the frequency band $f\in \left[0.5,6\right]$ GHz before and after leavening and evaluate statistically their consistency, repeatability, and reliability.

## 2. Materials and Methods

### 2.1. Microwave Dielectric Spectroscopy

#### 2.1.1. Sample Preparation

The Carasau bread doughs analyzed in this study were prepared following a protocol like that used in previous research [19,20,21,22]. The CB dough samples were prepared using commercial semolina, distilled water, fresh brewer’s yeast (*Saccharomyces cerevisiae*, Lievital, Parma, Italy), and commercial sea salt (Selex, Milan, Italy). Taking semolina as the reference component, the relative percentages of water (*W*), yeast (*Y*), and salt (*NaCl*) were defined based on its weight so that the nominal recipe requires 300 g of semolina, 150 g of distilled water, 4.5 g of NaCl, and 4.5 g of yeast. In this work, a single batch of semolina wheat was used to prepare all samples. The water temperature was set equal to 26 °C. Dough was prepared using a Sana Smart Breadmaker (SANABMS, Sana S.r.o., Ceske Budejovice, Czech Republic) machine for 20 min at 88 rpm and at room temperature. The doughs were prepared to have an approximate size with a diameter of 5 cm and a height of 5 cm. Ten identical CB dough samples were prepared.

#### 2.1.2. Microwave Measurements

Food materials have a unique dielectric signature and electromagnetic properties determined by the water content and the composition. Dielectric properties at MW frequencies are expressed in terms of the relative complex permittivity $\epsilon =\epsilon^{\prime }-j\epsilon^{\prime \prime },$ being ϵ∈C and $j=\sqrt{-1}$. The term $\epsilon^{\prime }$ is called the real part and accounts for the stored electric energy; i.e., it measures the polarizability and strength of the electric field inside of a given material. On the other hand, the term $\epsilon^{\prime \prime }$ is defined as the imaginary part that accounts for free-charge movements and polarization losses [19]; i.e., it is a measure of how much energy the electric field loses in the interactions with the external field, accounting for absorption and energy dissipation mechanisms. Depending on the variations of $\epsilon^{\prime },\;\epsilon^{\prime \prime }$ with the frequency of the applied signal f, relevant information about food materials can be derived.

These MW dielectric properties of CB dough samples are measured using an open-ended coaxial probe (OCP) dielectric assessment kit (DAK 3.5, Speag, Gilching, Germany) probe, for a frequency range from 500 MHz to 6 GHz connected to a vector network analyzer (VNA) Rohde & Schwarz ZNB 8 (9 KHz–8.5 GHz). The frequency range adopted in this study, compared to the state of the art, covers the industrial, scientific, and medical (ISM) bands of 0.915 GHz, 2.45 GHz, and 5.8 GHz [19,20,21,22]. In OCP MW DS measurements, random, systematic, and drift errors can affect the reliability, as well as sample geometry and other factors [19,20,21,22]. This is why the OCP measurement system was calibrated using standard procedures (i.e., open, short, and load—OSL), and the load temperature was monitored using a PT100 thermometer to ensure the reliability of results. Deionized water, with a temperature monitored to within ±0.05 °C, was used as the load standard.

After calibration, the OCP was then positioned in direct contact with the sample to collect reflection coefficient data, which was converted into complex permittivity values using the DAK software (V3.0.6.34). Then, during the measurements, the electrically thick doughs (5 cm × 5 cm) were put in direct contact with the OCP, and ten measurements per sample were taken to ensure reliable results, and the room experimental conditions (e.g., temperature, humidity) were carefully monitored. The CB dough samples’ MW dielectric properties are acquired immediately after mixing t=0 min and after t=40 min of leavening in a covered sample holder. Error sources, including VNA noise and sample inhomogeneity, were addressed through calibration and considering the combined uncertainty from drift and random errors [19,20,21,22,23,24].

#### 2.1.3. Environmental Parameters

To monitor the environmental parameters that can affect dough preparation, leavening, and the MW DS measurements, the tested and validated wireless sensors network (WSN) node from [25,26,27] was used. In brief, a GY-BME/BMP280 (Yi Yue, Shenzhen, China) sensor module for temperature (T, in °C), humidity (H, %) and pressure (P, in bar) was integrated in a dedicated printed circuit board (PCB) designed using Eagle Autodesk (EDA Solutions, Fareham, UK) and manufactured with LPKF Protomat C100 HF (LKPF, Garbsen, Germany), equipped with a logical level converter of 5–3.3 V. The acquired data are all digital and can be easily handled by a Raspberry Pi unit. The Raspberry units transmit wirelessly using Wi-Fi the collected data to a server. Data are organized and managed using the open-source database Elasticsearch (Elasticsearch, Mountain View, CA, USA), stored in “.json” format and handled with a GUI based on the free tool called Kibana (Elasticsearch, USA). The WSN node acquired continuously ($f_{s}≃1$ ms) the environmental data in the room where the doughs are mixed, kneaded, and tested with the OCP. By relying on the time stamps, a subset of environmental data was extracted and co-registered with the OCP measurement times.

### 2.2. Statistical Analysis

To use MW DS as a tool to empower the industrial production of Carasau bread, hence correlating the dielectric signature with the processing parameters and product features, it is fundamental to understand the trustability and reliability of the measurements for different samples. In particular, since leavening is considered to be the most crucial step for determining the product quality before baking [1,2,3,4,5,6,7,8,9,10,19,20,21,22], the MW DS measurements should be analyzed before and after leavening in [19]. This is why, in this work, an in-depth statistical analysis on the 10 dough samples and 10 measurements per dough are performed. In particular, the following methodology will be applied to (i) each of the ten dough samples, treated as a single group, and to (ii) the 100 measurements considered as a population. By performing the statistical analysis in these two conditions, a clear understanding of how the statistical size would impact the knowledge of MW properties of CB doughs will be achieved.

#### 2.2.1. Descriptive Statistics

To evaluate whether the MW DS spectra of the different samples present a different morphology over frequency, Pearson’s correlation coefficient (R), at each frequency f, was evaluated as follows (for i≠j).

Besides the morphological similarities, the MW spectra data for the 10 measurements of the 10 samples were processed to derive the mean $\mu_{\epsilon }$ for a given frequency f as [23]

(1) $$\mu_{\epsilon }\left(f\right)=\frac{1}{N}\sum_{i=1}^{N}\epsilon_{i}\left(f\right),$$

And the standard deviation $\sigma_{\epsilon }$, to account for data dispersion, as [23]

(2) $$\sigma_{\epsilon }\left(f\right)=\sqrt{\frac{1}{N-1}\sum_{i=1}^{N}{\left[\epsilon_{i}\left(f\right)-\mu_{\epsilon }\left(f\right)\right]}^{2}\;\;}.$$

The dielectric spectra of CB doughs before and after leavening are also analyzed in terms of outliers, and the means and standard deviations are compared.

Then, we performed a z-score normalization of the complex permittivity data ($\epsilon_{\mathrm{norm}}$) by taking the difference between the permittivity and the mean value, divided by the standard deviation [28,29]. In mathematical terms

(3) $$\epsilon_{\mathrm{norm}}\left(f\right)=\frac{\epsilon \left(f\right)-\mu_{\epsilon }\left(f\right)}{\sigma_{\epsilon }(f)}.$$

From the normalization, if $\epsilon_{\mathrm{norm}}>0$, the single MW DS measurement at a given frequency is higher than the mean, whilst if $\epsilon_{\mathrm{norm}}<0$, it is lower. If $\epsilon_{\mathrm{norm}}$ is close to zero, then the dielectric permittivity value tends to be close to the mean (i.e., $\epsilon \left(f\right)≃\mu_{\epsilon }(f)$). As the standard deviation of a given measurement set lowers, the value of $\epsilon_{\mathrm{norm}}$ increases.

The discussed figures of merit are not enough to elucidate completely the MW DS data. Therefore, we evaluate the coefficient of variation (CV) in percentage (%) expresses the relative variation compared to the average, i.e., [30]

(4) $$CV=100\cdot \frac{\sigma_{\epsilon }}{\mu_{\epsilon }}.$$

A low CV value, below 5%, indicates high repeatability [30,31]. The CV is computed before and after leavening for each frequency f.

#### 2.2.2. Kernel Density Estimation

Since the distributions of the complex dielectric permittivity values are unknown, we performed the kernel density estimation (KDE) and applied the Parzen–Rosenblatt window method [32,33,34]. This non-parametric method allows estimating the probability density function of a random variable based on kernels as weights, especially in situations where inferences about the population must be made based on a finite data sample, as in this case. In other words, the kernel distribution (K) is a nonparametric representation of a probability density function (pdf) of a random variable, herein ϵ(f). The use of this approach ensures avoiding making assumptions about the distribution of the data. Therefore, for any values of $\epsilon^{\prime }$ and $\epsilon^{\prime \prime }$ the kernel estimator is given by [32]

(5) $$F_{B}\left(\epsilon \right)=\frac{1}{N_{m}B}\sum_{j=1}^{N_{m}}w_{j}K\left(\frac{\epsilon -\epsilon_{j}}{B}\right)$$

where $N_{m}$ is the number of samples, B is the bandwidth, and $w_{j}$ is the j-th sample. In this analysis, the normal kernel was used [32]

(6) $$K=\frac{1}{\sqrt{2\pi }}e^{\frac{-{\left(\epsilon -\epsilon_{j}\right)}^{2}}{B}}.$$

This formalism allows us to derive relevant information about the distribution of the permittivity of the samples.

The descriptive statistics, based on the discussed figures of merit, is performed (i) considering the samples as 10 different sets of measurements and (ii) considering all 100 measurements as a single set and population, both before and after the leavening.

## 3. Results and Discussion

This work aims at assessing the MW spectra for standard Carasau bread doughs before and after leavening, focusing on the consistency, repeatability, and reliability using statistical methods.

**Figure 1:**
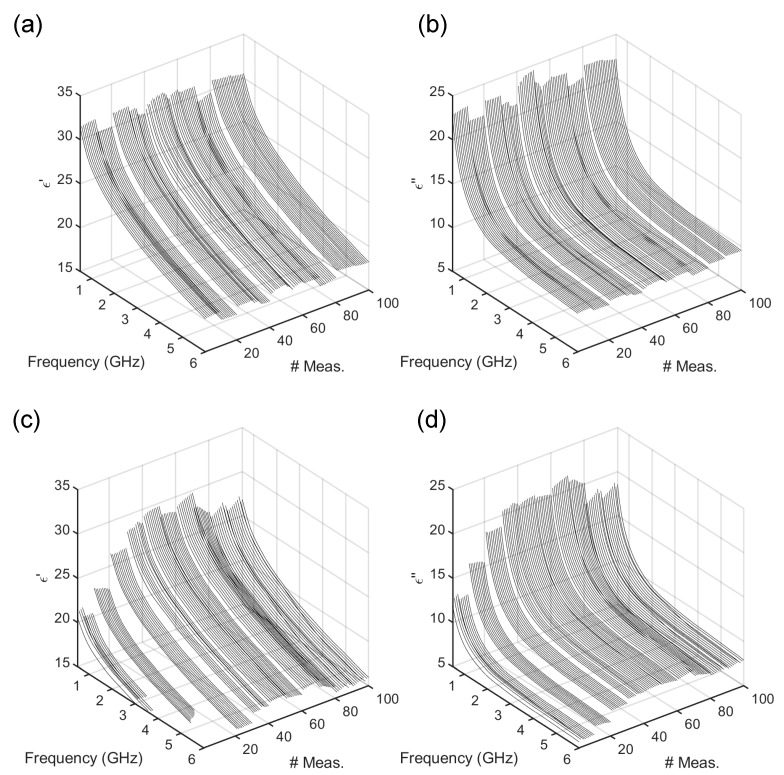
Dielectric permittivity data versus frequency (GHz) and no. of measurements before dough leavening (t=0 min) in terms of the (**a**) real part (ϵ′), (**b**) imaginary part ($\epsilon^{\prime \prime }$), and after leavening (t=40 min), in terms of (**c**) real part (ϵ′), (**d**) imaginary part ($\epsilon^{\prime \prime }$).

The MW DS study was performed replicating and manufacturing 10 different samples of standard CB doughs and measuring 10 times their spectra. These samples are known to present stickiness ranging between 0.02 and 0.30 Ns and hardness between 0.02 and 0.1 N [5] and complex viscosity values between 1000 and 10,000 Pa⋅s [6]. The level of dielectric losses observed in CB dough spectra in the γ-relaxation region at MW is correlated to a high compliance (~2.5$\cdot 10^{-4}$ 1/Pa) [10]. The dough compliance accounts, in turn, for the ratio of deformation and applied stress, are strictly correlated to the dough texture [10]. Therefore, the dielectric losses are well correlated with the rheological and texture properties of the CB doughs. The complete set of measurements in terms of the real (ϵ′) and imaginary ($\epsilon^{\prime \prime }$) parts before and after leavening is given in Figure 1. From Figure 1 it can be noticed that before leavening, the real and imaginary parts of the dielectric spectra show curves with similar trends but slightly different values. On the other hand, from Figure 1, considering the post-leavening condition, the spectra exhibit larger variations. In particular, after leavening, lower values in the imaginary part of the dielectric permittivity are observed, thus being associated with lower compliance (~1$\cdot 10^{-4}$ 1/Pa) [10], resulting in larger viscosities [6], so that the stickiness decreases and the hardness increases [5].

**Figure 2:**
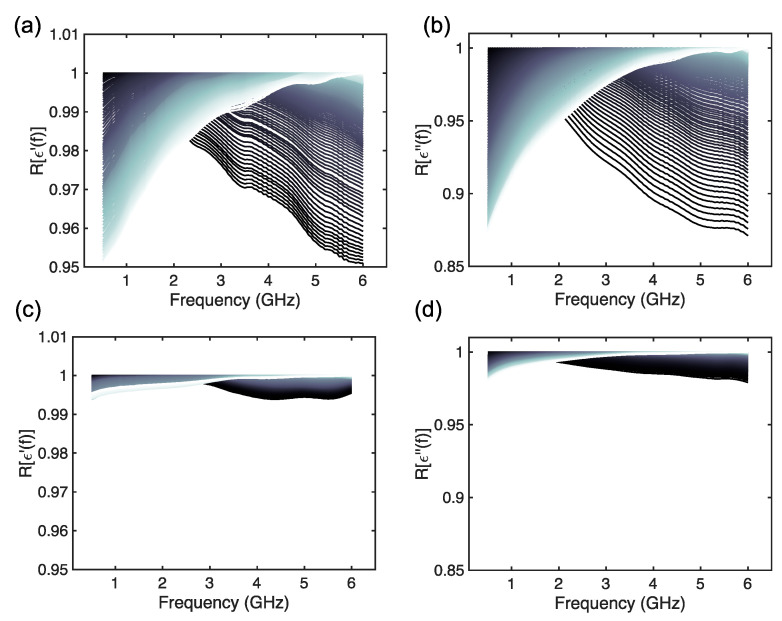
Pearson’s correlation coefficient (R) for the dielectric permittivity of Carasau bread dough data versus frequency (GHz), before dough leavening (t=0 min) for the (**a**) real part (ϵ′) and (**b**) imaginary part ($\epsilon^{\prime \prime }$), and after leavening (t=40 min) for the (**c**) real part (ϵ′) and (**d**) imaginary part ($\epsilon^{\prime \prime }$).

Considering the curves shown in Figure 1, Pearson’s correlation coefficient was evaluated, and the findings are reported in Figure 2. It can be noticed that the morphological similarity and trends are very high before (R>0.85) and after the leavening (R>0.99), thus suggesting that the observed dielectric permittivity dispersion, independently from the values, is coherent for all 100 measurements and 10 samples. Anyway, the correlation coefficient is not enough to guarantee a reliable metric for assessing the consistency of the MW DS measurements of CB doughs. The differences across these MW spectra of such relevant food material must be studied quantitatively.

**Figure 3:**
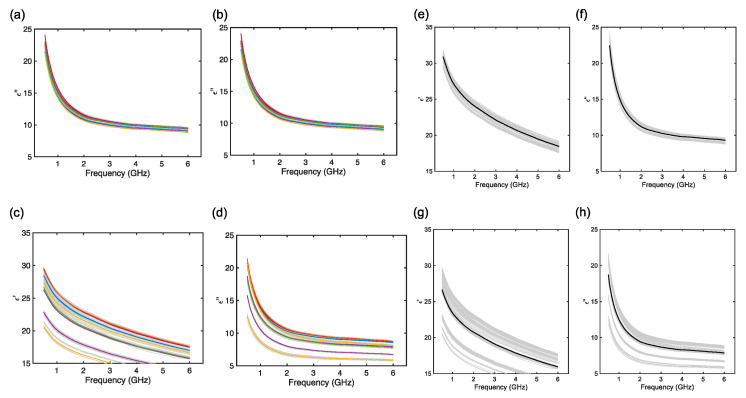
MW DS spectra before leavening (t=0 min): (**a**) real part of the permittivity (ϵ′) and (**b**) imaginary part of the permittivity ($\epsilon^{\prime \prime }$) with the ten means for each sample overimpressed. MW DS spectra after leavening (t=40 min): (**c**) real part of the permittivity (ϵ′) and (**d**) imaginary part of the permittivity ($\epsilon^{\prime \prime }$) with the ten means for each sample overimpressed. MW DS spectra before leavening (t=0 min): (**e**) real part of the permittivity (ϵ′) and (**f**) imaginary part of the permittivity ($\epsilon^{\prime \prime }$) with the mean calculated from all measurements. MW DS spectra after leavening (t=40 min): (**g**) real part of the permittivity (ϵ′) and (**h**) imaginary part of the permittivity ($\epsilon^{\prime \prime }$) with the mean calculated from all measurements.

Therefore, the mean was evaluated using Equation (1) and computed for each 10 measurements for a given dough sample and, also, for the complete set of measurements. The spectra are reported in Figure 3. The figures report the two different ways of evaluating the mean of the measurements, i.e., considering each sample as a different set and considering the complete 100 measurements as a single set. From Figure 3 it is possible to identify interesting features of the MW DS spectra of CB doughs before and after leavening. Before leavening, the permittivity values at each frequency are very close for all samples, and the means appear to be close, both for the real and imaginary parts of the permittivity (Figure 3a,b). Therefore, considering Figure 3e,f, the mean values of the permittivity are very close to the measured values (~3%), that is very well within the measurement error and uncertainty [19,23]. This can indicate that MW DS can be a precise tool for assessing the features of CB doughs. However, by observing Figure 3c,d, after leavening, the real and imaginary parts of the dielectric spectra present very different values and well-separated mean curves. Considering Figure 3g,h, the mean differs up to ~25% from the measured values. Therefore, from these findings, it is possible to infer that relevant changes in the dielectric permittivity spectra have occurred during the leavening. This was observed in [19] and attributed to the loss of water and increase in the air content due to the yeast and fermentation reaction. However, with respect to the results from [19], we must notice that the variations in the dielectric permittivity are not constant and not the same for all the 10 samples. In other words, apparently, the complex and non-linear process of leavening is decreasing the complex permittivity of CB doughs, but not in the same way for all the tested samples. The implications of these findings are already relevant since, despite the morphological trend, they may suggest that MW DS can also be used as a tool to evaluate the leavening process, thus supplying CB manufacturers with an engineered tool. Anyway, this semi-qualitative analysis is not enough and demands a refined investigation.

**Figure 4:**
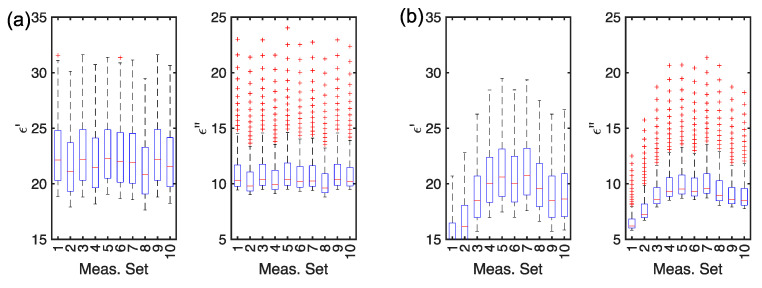
Boxplots of (**a**) the permittivity real part ($\epsilon^{\prime }$) and imaginary part ($\epsilon^{\prime \prime }$) before leavening and of the (**b**) the permittivity real part (ϵ′) and imaginary part ($\epsilon^{\prime \prime }$) after leavening (t=40 min).

To this aim, the boxplots of the real and imaginary parts of the permittivity, before and after leavening, are given in Figure 4. The boxplots are computed for all frequencies f. From Figure 4 it can be noted that the means are not far across the measurement sets before leavening and that the standard deviations are very similar. For the imaginary part of the permittivity, outliers have been identified, but they are uniformly distributed.

**Figure 5:**
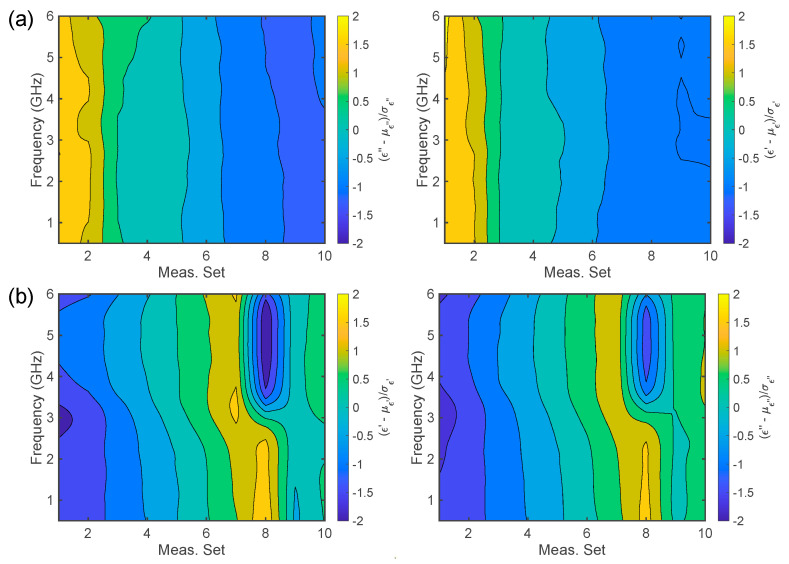
Normalized complex dielectric permittivity Carasau bread dough data versus frequency (GHz) for 10 clustered measurements: (**a**) before dough leavening (t=0 min) and (**b**) after leavening (t=40 min), for the real and imaginary parts of the dielectric permittivity.

To further investigate the differences in the dielectric signature of CB dough samples, the z-score normalized data were investigated. Each subset of 10 measurements is compared to its mean and standard deviation (see Figure 4). The findings are given in Figure 5. For the clustered samples, before leavening (Figure 5a), it can be noticed that the first and the last samples have $\epsilon^{\prime }$ and $\epsilon^{\prime \prime }$ very far from the mean at all frequencies f. On the other hand, after the leavening, the first four samples show $\epsilon^{\prime }$ and $\epsilon^{\prime \prime }$ below the average, especially for f∈[4.5, 5.5] GHz, whilst sample number 8 presents a more complicated variation in the z-score vs. frequency, with a minimum in f∈[4.5, 5.5] GHz, and a peak for f<3 GHz. This behavior may suggest that after leavening, the dough samples underwent significant changes that affected their spectra and the permittivity distributions.

**Figure 6:**
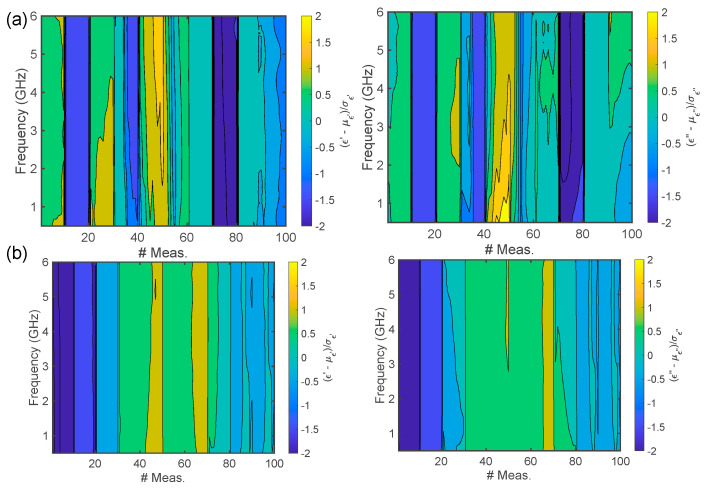
Normalized complex dielectric permittivity Carasau bread dough data versus frequency (GHz) for all 100 measurements: (**a**) before dough leavening (t=0 min) and (**b**) after leavening (t=40 min), for the real and imaginary parts of the dielectric permittivity.

In Figure 6, the z-score contour plot over frequency and the number of measurements is presented. With respect to Figure 5, it is worth specifying that the mean and standard deviation are computed over the whole 100-measurement set. In Figure 6a it is possible to observe that, at t=0 min, the complex permittivity reaches values below the mean for measurements 10–20, 30–40, and 70–80, whilst it is higher for measurements 40–50. On the other hand, after leavening, this situation drastically changes, and dips in the z-score are observed for measurements 1–20, whilst the occurrence of permittivity values higher than the mean are less frequent. By comparing the z-score from Figure 6 with that from Figure 5, it can be noticed that the deviations from the mean are uniform with respect to frequency.

**Figure 7:**
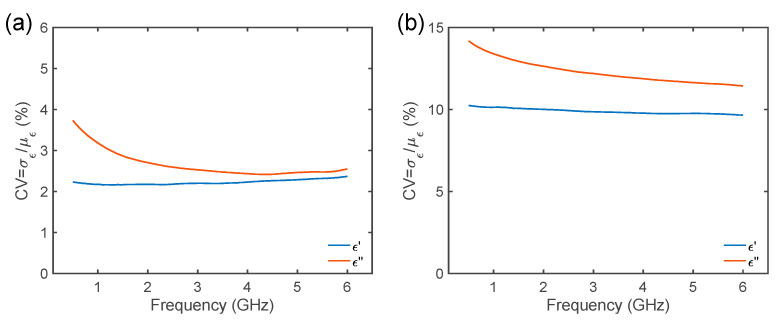
Coefficient of variation (CV) for the complex dielectric permittivity Carasau bread dough data versus frequency (GHz), (**a**) before dough leavening (t=0 min) and (**b**) after leavening (t=40 min).

Therefore, by analyzing the z-scores in Figure 5 and Figure 6, it can be inferred that relevant differences may occur during the different measurements and after leavening. To further study this behavior seeking a quantitative figure of merit, the CV was investigated (see Equation (4)). In Figure 7, the CV of the MW DS measurements was quantified over frequency before and after leavening. From Figure 7a it can be seen that the coefficient of variation for both the real and imaginary parts of the permittivity is below the threshold of 5%, thus indicating that the performed measurements are robust and consistent. In particular, the MW DS data are more reliable for the real part of the permittivity ($\epsilon^{\prime }$), whilst for the imaginary part ($\epsilon^{\prime \prime }$) the low-frequency (f<2 GHz) data show higher variability. On the other hand, in Figure 7b, after leavening CV>5%, reaching 10% for all frequency for ϵ′ measurements and ~13.5% for $\epsilon^{\prime \prime }$ for all frequencies. If the standard deviations are small, the coefficients of variation are low (<5%), and we can conclude that the measurements are highly repeatable. However, if the variability is high, sources of instrumental or experimental error are generally considered, but, herein, food materials are involved. Therefore, again, the physical features of the CB dough samples after leavening, which is a complex non-linear bio-chemical phenomenon, lead to significant differences across samples and measurements. From these findings it is possible to deduce that the dielectric response of these food materials after leavening demands further investigation.

To assess, from a statistical point of view, the nature of the MW DS measurements on CB dough samples, a non-parametric analysis based on KDE was performed. This analysis was performed for the 10 clusters and for the 100 measurements.

**Figure 8:**
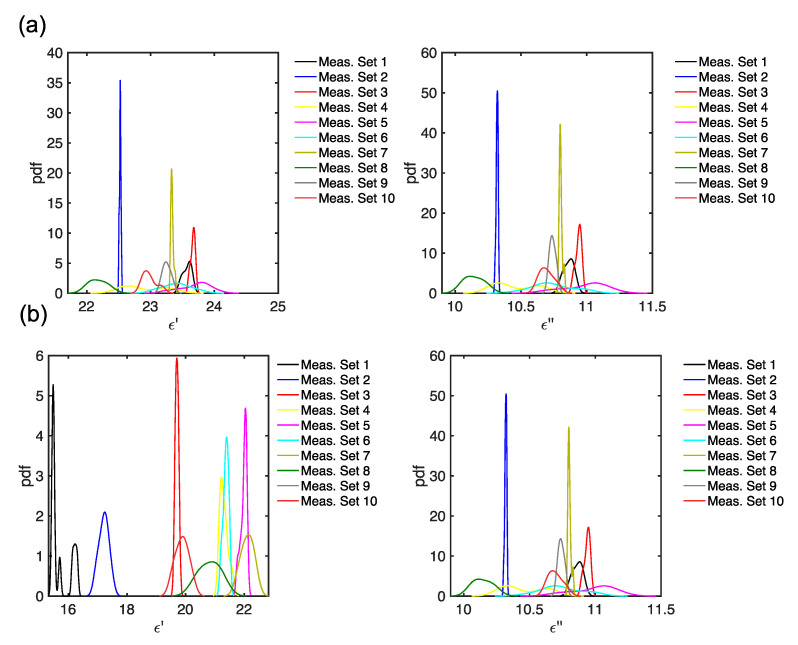
KDE for the complex dielectric permittivity Carasau bread dough data for the 10 measurement sets, for f=2.45 GHz, (**a**) before dough leavening (t=0 min) and (**b**) after leavening (t=40 min).

The KDE for f=2.45 GHz for the 10 measurement sets is reported in Figure 8. Interestingly, the probability density functions for the 10 different clusters are not gaussian and normal for both the stored electric energy ($\epsilon^{\prime }$) and the polarization and conduction losses ($\epsilon^{\prime \prime }$), both before and after leavening. Therefore, using conventional statistical methods that assume normal distributions (e.g., ANOVA, etc.) cannot be employed on these MW DS data. Furthermore, by comparing Figure 8a with Figure 8b, it is possible to notice that the estimated distributions modify for most samples after the leavening (e.g., see the blue curve for sample #2 and the black curve for sample #10 in Figure 8).

Anyway, considering the 10 samples as independent and separate may not be the best choice; therefore, to complete the study, the entire 100 measurements should be considered.

**Figure 9:**
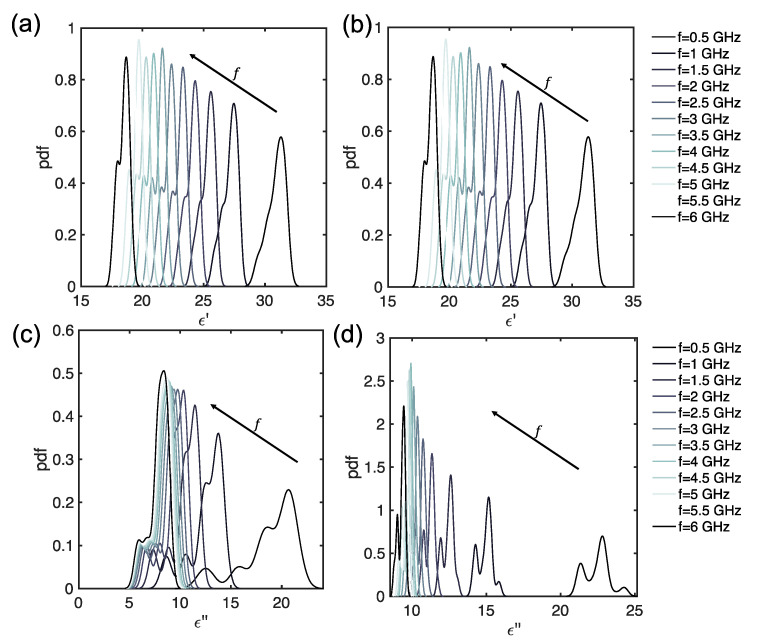
KDE for Carasau bread dough data, for different frequencies (GHz): (**a**) $\epsilon^{\prime }$ before dough leavening (t=0 min), (**b**) $\epsilon^{\prime \prime }$ before dough leavening (t=0 min), (**c**) $\epsilon^{\prime }$ after leavening (t=40 min), and (**d**) $\epsilon^{\prime \prime }$ after leavening (t=40 min).

In Figure 9 the probability density functions KDE for the 100 measurements and for various frequencies are reported. It can be noticed that, considering a given frequency value, pronounced peaks can be observed for ϵ′ and $\epsilon^{\prime \prime }$, before leavening (Figure 9a,b). However, as frequency increases, a shoulder appears in the KDE. After the leavening, the KDE presents more complicated behavior, since several peaks and shoulders are evident at any frequency for $\epsilon^{\prime }$ and $\epsilon^{\prime \prime }$, thus showing the larger variability of the MW DS measurements.

**Figure 10:**
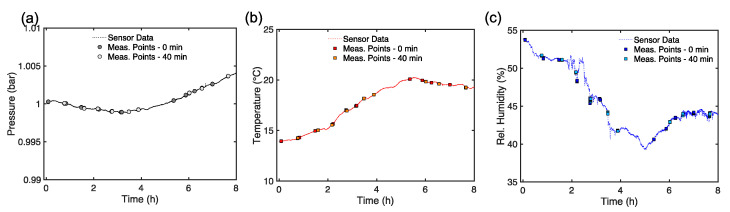
(**a**) Room pressure (in bar) over measurement time (in h). (**b**) Room temperature (in °C) over time. (**c**) Relative humidity (in %) over time.

**Figure 11:**
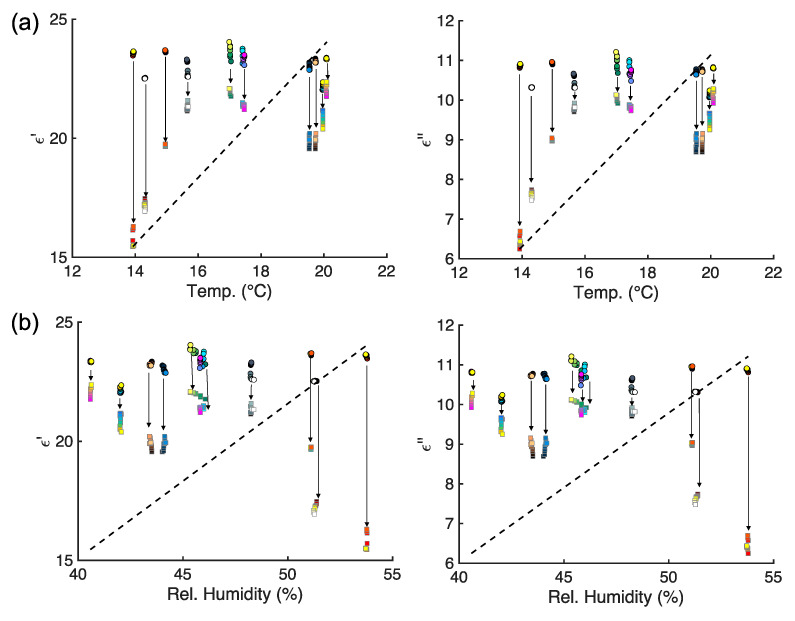
Scatter plots of the (**a**) real part ($\epsilon^{\prime },\;$ **left**) and imaginary part ($\epsilon^{\prime \prime }$, **right**) of the permittivity versus room temperature (in °C). (**b**) Real part ($\epsilon^{\prime },$ left) and imaginary part ($\epsilon^{\prime \prime }$, right) of the permittivity versus relative humidity (in %). Circles represent dielectric data at t=0 min and squares represent dielectric data at t=40  min. The 10 different colormaps represent the measurement batches. The black dashed line represents the smoothing function.

From the analysis performed so far, we have highlighted that the MW DS of CB doughs can reliably estimate the food material characteristics before leavening, with limited variability, but, after leavening, the MW DS data presents complicated patterns and statistics, challenging the potential use of high-frequency electromagnetic devices to supply the industrial production and automation of this traditional and valuable food material. Most of the findings suggested that relevant differences in the analyzed figures of merit arise after the leavening phase. Leavening, from an MW DS point of view, calls for an increase in air content in the dough matrix, with a decrease in water (free or bounded) amount and modifications of the gluten network [19]. During the leavening mass and heat transport phenomena, bound and free water play a major role in determining the organoleptic properties. Indeed, during leavening, the production of air bubbles and loss of moisture and water content are ruled by physical laws (e.g., diffusion, thermal conduction, and convection, etc.) that depend upon the environmental parameters. As a matter of fact, the dielectric signature of the CB reflects these physical changes. Therefore, it would be reasonable to ask if the variability of the dielectric properties observed before and after the leavening may be tracked back to variations and changes in the environmental parameters (i.e., T, P, H). Given the statistical analysis performed so far, and provided that these environmental parameters have been monitored during the measurements, it is possible to execute an additional analysis to answer this question. Even though it must be highlighted that the temperature of the doughs was about 26 °C, with fluctuations very close to the PT100 accuracy, for all samples after mixing, and that, after leavening, the dough samples’ temperature was close to ~28.3 ± 0.8 °C, a more detailed analysis is in order. In Figure 10 the curves for the pressure (in bar), the room temperature (in °C), and the relative humidity (in %) are given. In Figure 10 the points show the precise moment in which the MW DS data were taken. From Figure 10a it can be seen that the pressure is almost constant. On the other hand, it can be noticed that temperature increases of about 5 °C and that relative humidity has a drop of ~15% during the measurements. Hence, time may be a dimension that can be relevant, as highlighted preliminary in [19]. Therefore, assuming that ambient pressure can be neglected, we analyzed how the MW DS data versus temperature and relative humidity are distributed, considering the time variation, as reported in Figure 11. It is possible to observe that there is not a linear correlation between the permittivity data and temperature (Figure 11a) or relative humidity (Figure 11b), both before and after leavening. Therefore, results from Figure 11 highlight that the evaluation of the influence of temperature and relative humidity variation on the dielectric permittivity spectra can be negligible, or, at least, demands for further and more structured studies.

The findings from Figure 11 show that more advanced and statistical methods that consider the heterogeneity of the distribution are needed to fully explain and interpret the complex interplay between MW dielectric spectra of CB doughs before and after leavening. In particular, methodologies such as regression discontinuity design, which is a quasi-experimental statistical technique used to identify causal effects by exploiting a discontinuity in a treatment assignment variable [35], can be applied to the CB dielectric permittivity data that show a discontinuous shift at a specific processing condition (Figure 11) and may model the variation and estimate the causal impact of that processing stage. Therefore, our findings may pave the route for an innovation in the bakery industry, especially the CB, fostering its advancement and automation [36].

## 4. Conclusions

This study provides a systematic evaluation of the microwave dielectric spectroscopy (MW DS) measurements for Carasau bread (CB) dough characterization, focusing on repeatability, reliability, and consistency. The results indicate that while MW DS exhibits strong spectral correlation across samples and maintains a coefficient of variation below 5% before leavening, statistical analyses such as z-score and kernel density estimation reveal significant heterogeneity in dielectric data distribution, particularly after leavening. Despite excluding the influence of pressure, temperature, and relative humidity, variability across samples remains evident, highlighting the complexity of CB dough properties. These findings underscore the need for further refinement of MW DS methodologies to enhance robustness for industrial applications. To overcome limitations related to specificity and sensitivity, future research should explore additional factors influencing dielectric properties and optimizing MW DS protocols to improve measurement precision in food quality monitoring. Furthermore, efforts should be devoted to investigating the correlation between MW DS measurements and other physical characterization methods, such as texture analysis or rheology, so that such a joint collaborative and multidisciplinary effort can lead to the development of the industrial production of a valuable traditional food product.

## Data Availability

The data presented in this study are available on request from the corresponding author.

## Abbreviations

The following abbreviations are used in this manuscript:

CB	Carasau Bread
CV	Coefficient of Variation
DAK	Dielectric Assessment Kit
DS	Dielectric Spectroscopy
FB	Flat Bread
MW	Microwaves
NMR	Nuclear Magnetic Resonance
OCP	Open-ended Coaxial Probe
VNA	Vector Network Analyzer
WSN	Wireless Sensors Network
